# Collection, genotyping and virus elimination of cassava landraces from Tanzania and documentation of farmer knowledge

**DOI:** 10.1371/journal.pone.0255326

**Published:** 2021-08-17

**Authors:** M. E. Ferguson, S. Tumwegamire, C. Chidzanga, T. Shah, K. Mtunda, H. Kulembeka, B. Kimata, S. Tollano, M. Stephen, E. Mpayo, S. Mohamedi, S. Kasele, E. Palangyo, J. Armachius, A. Hamad Ali, K. Sichalwe, D. Matondo, F. Masisila, Z. Matumbo, B. Kidunda, A. C. Arati, R. Muiruri, F. Munguti, A. Abass, M. Abberton, G. Mkamilo

**Affiliations:** 1 IITA, Nairobi, Kenya; 2 IITA, Dar es Salaam, Tanzania; 3 The University of Adelaide, Glen Osmond, Australia; 4 TARI, Kibaha, Tanzania; 5 TARI, Ukiriguru, Tanzania; 6 TARI, Naliendele, Tanzania; 7 TARI, Hombolo, Dodoma, Tanzania; 8 ARI, Kizimbani, Zanzibar; 9 TARI, Tengeru, Arusha, Tanzania; 10 KEPHIS, Muguga, Kenya; 11 IITA, Ibadan, Nigeria; Sivas Bilim ve Teknoloji Universitesi, TURKEY

## Abstract

Cassava (*Manihot esculenta* Crantz.) has been a vital staple and food security crop in Tanzania for several centuries, and it is likely that its resilience will play a key role in mitigating livelihood insecurities arising from climate change. The sector is dominated by smallholder farmers growing traditional landrace varieties. A recent surge in virus diseases and awareness in the commercial potential of cassava has prompted a drive to disseminate improved varieties in the country. These factors however also threaten the existence of landraces and associated farmer knowledge. It is important that the landraces are conserved and utilized as the adaptive gene complexes they harbor can drive breeding for improved varieties that meet agro-ecological adaptation as well as farmer and consumer needs, thereby improving adoption rates. Here we report on cassava germplasm collection missions and documentation of farmer knowledge in seven zones of Tanzania. A total of 277 unique landraces are identified through high-density genotyping. The large number of landraces is attributable to a mixed clonal/sexual reproductive system in which the soil seed bank and incorporation of seedlings plays an important role. A striking divergence in genetic relationships between the coastal regions and western regions is evident and explained by (i) independent introductions of cassava into the country, (ii) adaptation to prevailing agro-ecological conditions and (iii) farmer selections according to the intended use or market demands. The main uses of cassava with different product profiles are evident, including fresh consumption, flour production, dual purpose incorporating both these uses and longer-term food security. Each of these products have different trait requirements. Individual landraces were not widely distributed across the country with limited farmer-to-farmer diffusion with implications for seed systems.

## Introduction

The clonally propagated crop cassava (*Manihot esculenta* Crantz), which originated around the rim of the Amazon Basin [1,2], was introduced to Tanzania in the 17^th^ and 18^th^ centuries. It is likely that this introduction occurred independently from the coast, through the trading ports on the islands of Zanzibar and Pemba, and from the West via Congo and Angola [3–5]. Due to its natural resilience and adaptation to most soils and fluctuating water balances, cassava has played a crucial role as a subsistence and food security crop, producing a source of concentrated carbohydrate when other crops may fail [6]. It is now the second most important food staple in Tanzania after maize in terms of production and *per capita* consumption [7].

Cassava is both an outcrossing species as well as being vegetatively propagated. Throughout this document the term “seed” refers to cassava stem cuttings which are the planting material of cassava. True seed is referred to as botanical seed. The vast majority of cassava in Tanzania is grown by smallholder farmers who recycle their own cuttings of traditional landrace cultivars [8] and recruit seedlings germinated from the botanical seed bank in the soil to supplement planted cultivars [9]. The soil seed bank is the natural storage of botanical seed in the soil or its’ surface after seed dispersal, which serves as a repository for the production of subsequent generations of plants to enable their survival [10]. A landrace can be defined as a dynamic population(s) of a cultivated plant that has historical origin, distinct identity and lacks formal crop improvement, as well as often being genetically diverse, locally adapted and associated with traditional farming systems [11]. Human and environmental selection pressures mold this diversity into valuable adaptive gene complexes that respond to prevailing agro-ecological conditions, biotic and abiotic stresses and cooking or organoleptic properties favored by consumers, while maintaining diversity [12,13]. This diversity is able to meet the varied needs of farmers and consumers and provides resilience to the system, an important element for the livelihood strategies of smallholder farmers.

Farmers often hold several generations of knowledge concerning the attributes of these landraces, and sometimes have specific reasons why they retain particular cultivars. In cassava in Uganda these preferences focused on culinary attributes, storability in the ground, early maturity and cooking quality [14]. Reasons for preserving landraces have been well documented in a range of crops and are summarized by Jarvis *et al*. [15].

Traditionally, in Tanzania, cassava roots are either consumed fresh (raw, boiled or fried) or are processed into flour and consumed as a stiff porridge (ugali). Varieties for fresh consumption taste sweet which indicates that they are low in cyanogenic glycosides, a compound that releases toxic hydrogen cyanide when chewed or digested and is associated with bitter cassava varieties [16]. Some sweet varieties can also be made into flour and are thus dual purpose. Bitter varieties are always processed into flour. During processing, volatile hydrogen cyanide gas is released, effectively de-toxifying the cassava. In Tanzania cassava is increasingly becoming a cash crop, exported as dry chips and being processed into cassava flour for a variety of uses and the baking industry [17,18]. It also has potential for use in local starch, brewing and ethanol production industries [19]. Increasing population pressure and demand for food, a surge in two virus diseases (cassava brown streak disease (CBSD) and cassava mosaic disease (CMD)) and their vector, the whitefly (*Bemisia tabacii*), and opportunities for commercialization have fueled efforts to distribute improved cassava varieties. The importance of these improved varieties is recognized for increased productivity and successful commercialization with the concomitant effects on income generation and improved livelihoods [20]. Indeed, projects such as Building an Economically Sustainable Seed System for Cassava in Tanzania (BEST Cassava) [21] focus on the rapid, large-scale distribution of improved, disease-resistant planting materials to farmers. Thus, biotic stresses and the adoption of improved varieties are accelerating the loss of landraces and associated farmer knowledge. It is critical that this genetic variability upon which breeding efforts depend, and the knowledge which drives the prioritization of attributes for ultimate successful cultivar adoption, is captured, preserved and utilised. This diversity is fundamental in trying to achieve global food security and sustainable development in the face of climate change and human population growth.

Unfortunately, Tanzanian landraces and their associated farmer knowledge are seriously under-represented in national and international germplasm collections [3,22]. Tanzanian landraces are possibly of particular importance due to the inter-specific breeding program that took place in Amani, Tanga, in the 1930s to 50s [23]. When this breeding program ceased, some of the breeding germplasm may have been incorporated into surrounding farms [24]. Indeed Kawuki *et al*. [22] found that cassava germplasm from Tanzania occupied a pivotal position within eastern, southern and central Africa. Recently the value of the cassava genebank at The International Center for Tropical Agriculture (CIAT) has been demonstrated through the uncovering of putative immunity to CBSD [25].

As a first step to conserve and characterize Tanzanian cassava landraces and farmer associated knowledge, germplasm collection missions were conducted, and knowledge documented. All landraces were genotyped to identify duplicates and a fingerprint obtained for future tracking purposes. Here we report on the collection missions, diversity of landraces and their associated farmer knowledge and on efforts to conserve them in perpetuity. It is anticipated that this will contribute to a gender-responsive, demand-led approach to cassava breeding that will result in the better adoption of new improved varieties [26,27].

## Materials and methods

### Collection of cassava landraces and associated farmer knowledge

**Fig 1:**
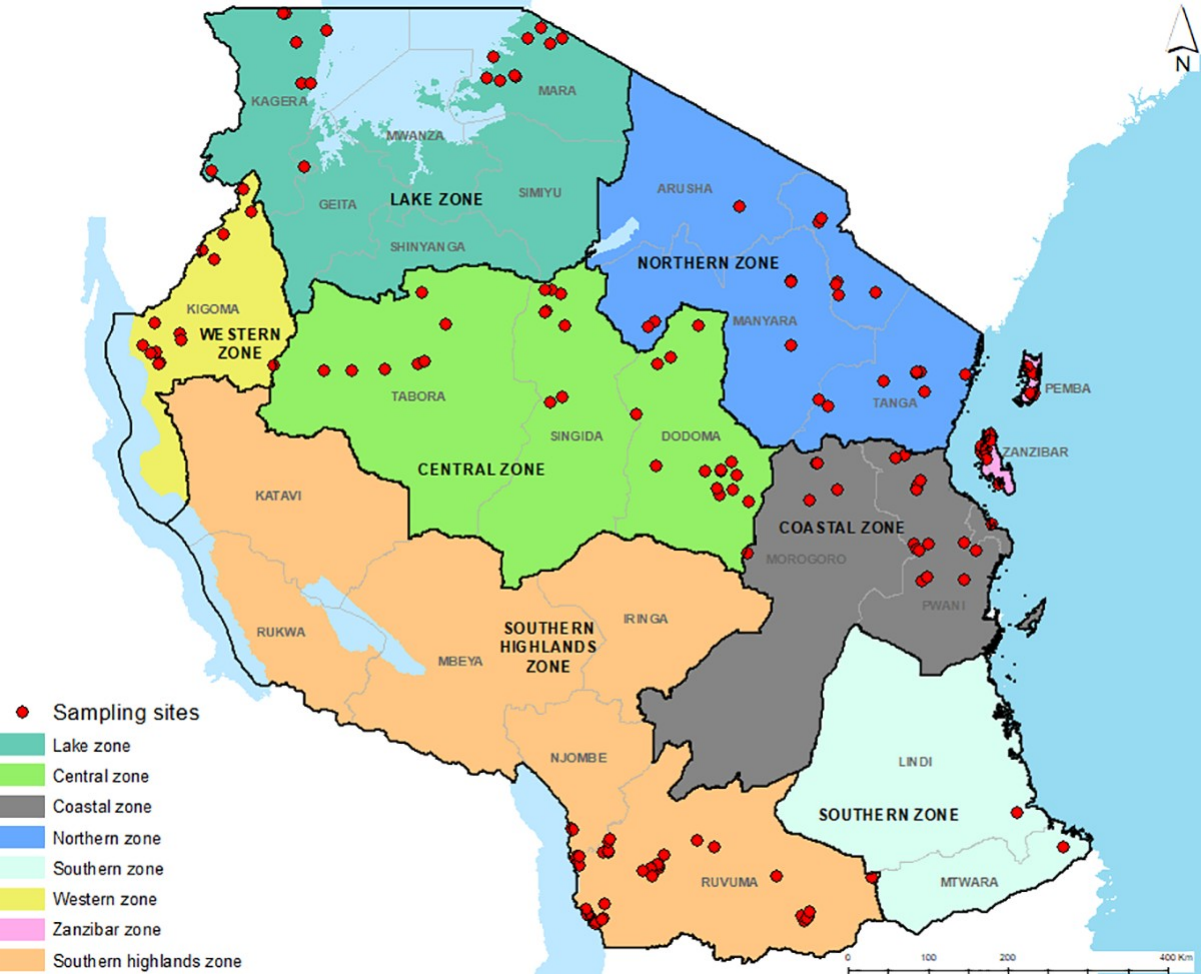
Location of 193 sites in Tanzania where cassava landraces were collected and key informant interviews conducted.

Sixty-six districts within Tanzania’s main cassava growing administrative zones were targeted for germplasm collection, namely; Lake, Western, Southern Highlands, Central, Northern, Coastal and Zanzibar Zones. The zone of Zanzibar includes the islands of Pemba and Unguja (Fig 1). Traditional cassava growing areas were prioritized as well as those areas particularly threatened by high disease pressure and with contrasting environmental characteristics. Collection missions and all surveys were undertaken by the Ministry of Agriculture in Tanzania (now Tanzanian Agricultural Research Institute (TARI)) in collaboration with the International Institute of Tropical Agriculture (IITA) from June 2016 to January 2017. All collections were carried out under the terms of the International Treaty of Plant Genetic Resources for Food and Agriculture (ITPGRFA) (http://www.fao.org/plant-treaty/en/). The collection team consisted of at least one cassava breeder/germplasm scientist and a social scientist.

In each district, a traditional custodian of crop diversity, or farmer known to grow a broad range of landraces was identified through local agricultural extension agents. That person was then asked about other farmers in the area keeping cassava landraces. Acquisition of farmers knowledge followed the ITPGRFA and the terms of the Convention on Biological Diversity including the Nagoya Protocol. These include prior informed consent. At each farm, three questionnaires were conducted which are detailed in Cox *et al*. [28] and were designed to capture farmer knowledge and preferences: (1) a ‘Key Informant Interview’ to profile each farmer, their farm and identify cultivars that they grew, (2) an ‘Individual Landrace’ form documented information on the history of the landrace, farmer’s experiences and preferences of the landrace, both of agronomy, response to biotic and abiotic stresses and cooking and eating properties, and (3) a more traditional germplasm collection form, designed to document morphological and agronomic descriptors of individual landraces, based on Fukunda *et al*. [29], and describe the collection environment.

Decision to collect germplasm from a farmer was based on the farmer information as well as the experience of the collector/germplasm scientist. Germplasm and its associated farmer information was collected if a landrace with a particular name had not been collected before or the landrace name had been collected before, but from a different Parish or area, and if the breeder identified any unusual or interesting characteristics. For the collection of the landraces, a stake or stakes from a single plant of each landrace to be collected was taken, clearly labeled, tied in bundles, and wrapped in newspaper or in a breathable sack so that nodes did not get damaged during transportation.

A unique questionnaire code that referred to a single key informant interview with a single person, was assigned to each interviewed farmer. This questionnaire code linked all collected knowledge and material(s) to one location and the individual farmer who provided it. It was also used to record and track this information in the database. A unique collector number was also assigned to each collected landrace. The unique collector number connected all farmer knowledge to the individual landrace that it described and was also used to record and track the individual landraces in the database and germplasm repository.

### Field gene bank

Within one week of collection, stakes from the Northern Zone, Southern Highlands Zone, Coastal Zone and the zone of Zanzibar, were planted in a field genebank at Naliendele Agriculture Research Institute (NARI), Mtwara, with a back-up repository in pots in a screen house at the Sugarcane Research Institute (SRI), Kibaha. Each clone was planted in a single row of five plants. Due to quarantine restrictions related to CBSD within the country, landraces collected in the Lake Zone and Western Zone, were planted in a screen house at the Lake Zone Agriculture Research and Development Institute (LZARDI), Ukiriguru.

### Database

A relational database with all the collected information from the questionnaires was created in MS Access and the three sections of the questionnaire entered as three tables (Key Informant Interview, Landrace Form and Germplasm Collection Form) linked to each other through the unique questionnaire codes and collector numbers. The database was queried to summarize information relating to the collected landraces, the farmers, their experiences, preferences and knowledge. Co-ordinates locating sampling sites were plotted on a map using ArcGIS 10.7 [30].

### Assessment of cooking quality and organoleptic traits

To further investigate cooking quality and organoleptic traits, 22 genotypes were selected from the Naliendele field genebank for on-station evaluation and laboratory analysis. Criteria for selecting genotypes for evaluation were (1) five plants were available, (2) the clone was unique from other clones being selected according to genotyping data, and (3) according to the key informant interview, the clone was preferred, they wanted to grow more of that cultivar and usually it was sweet and had good cooking qualities. BKP13 used specifically for flour was included as a check/control. The following methodologies were used for cooking quality and organoleptic traits:

*Time to cook*: Two 4cm cores of two roots per plant of two plants were peeled and placed in 2.5 litres of boiling water in a 4 litre aluminium pot. Cooking was done on a traditional ‘jiko’ stove with charcoal. Root cores were tested at 10 min after dropping in boiling water, and every minute after that to determine if they were cooked. This was be done by penetrating the side of the root using a toothpick. Time taken for each sample to cook was recorded in minutes.

*Softness/firmness*: Each cooked root core was poked three times on three random spots on the root with a toothpick and scored on a scale of 1-3 where 1 = soft, 2 = intermediate; 3 = hard.

*Softness/Mealiness/Taste*: Once declared ‘cooked’, each root core was partitioned into three parts with each part being given randomly to each of three panelists to evaluate. Softness was recorded by each panelist after chewing the boiled root on a scale of 1-5 where 1= Extremely soft, 2= Soft, 3= Neither soft nor hard, 4=Hard and 5=Extremely hard. The most common score was recorded. Mealiness was scored based on a scale described by Ngeve [31] of 1-3, where 1= Not mealy at all-roots hard and unchewable; 2=fairly mealy-cooked roots looking watery (‘glassy’), a bit soft but hard to chew; 3= very mealy-roots soft and floury when broken open and chewable. Photos were taken of boiled roots. Taste was scored on a scale from 1 to 3 where 1 = sweet, 2 = intermediate and 3 = bitter.

#### Laboratory assessment of CNP and softness

Cores of 4 cm thick of each of two roots per plant of three plants per clone were placed in a plastic bag in a cool box and transported to the IITA Food lab in Dar es Salaam for estimation of cyanogenic potential (CNP) and post-cooking softness. CNP was assessed according to Essers *et al*. [32] and post-cooking softness using P/MT Magnus-Taylor pressure probe (penetrometer) using peak force (g) and positive area (g/sec).

### SNP genotyping

SNP genotyping was used as the basis to identify duplicate samples and assess diversity among the collected landraces. Young fresh leaf samples were collected in 96-well format from sprouted cassava stakes in the screen house at SRI and at LZARDI and preserved with silica gel. On reaching the laboratory leaf samples were dried in a freeze drier before grinding using a Genogrinder (SPEXSamplePrep), and DNA extracted using a modified Dellaporta *et al*. [33] method. The main modification was a x15 reduction in scale which made it unnecessary to filter the supernatant in Step 5. To aid in the identification of landraces, 35 ‘reference landraces’ from Tanzania (24), Malawi (4), Kenya (2) and Mozambique (5) were included (S1 File) and 21 common cultivars (S2 File). ‘Reference landraces’ refer to well-characterised landraces of known identity to be used as a reference panel for possible identification of unknown collected landraces, based on DNA fingerprinting. ‘Collected landraces’ are those that were collected during the collection missions. These reference samples included nine cultivars and 15 landraces that had been well characterized as part of a 5CP project and were available *in vitro* [34].

Biological and technical replicates were included to determine the best cut-off for defining a unique variety. There is always some degree of error in genotyping and there may be a degree of variation between individual plants of the same clone derived from mutation, particularly when they are collected from distant locations. Including biological and technical duplicates in the study can help define whether two samples should be considered the same clone or different clones. Seven biological replicates (different plants of the same variety) were included from three reference landraces Albert, Kiroba and Nachinyaya, and two biological replicates from the breeding line NDL2003/031. Two technical replicates (samples from the same plant) were included from five reference landraces (Hombolo, Kalinda, Limbanga, Mreteta and Salanga), and four reference breeding lines (Pwani, NDL2003/111, UKG2009/52, UKG2009/74). In addition, two technical replicates were included for two of the collected landraces Horti_35 and SMS23, and three technical replicates of the collected landrace EPM46. The quality and quantity of DNA was assessed using Nanodrop, and high-quality DNA of standard concentration sent to Diversity Array Technology (DArT), Canberra, Australia, for high density SNP genotyping using DArTSeq.

#### Data analysis

Allele calls from DArTSeq were converted to allele dosage format for downstream analysis. SNP data was filtered to exclude all SNPs with more than 5% missing data, together with all monomorphic SNPs (all sampled genotypes have the same nucleotide(s)), and any SNP with a minor allele frequency less than 0.05. Any genotype with more than 5.5% missing data was also deleted. Missing data was not imputed due to the diversity of the samples.

To identify duplicates a distance matrix was generated using in PLINK (ver. 1.9) in R (ver. 4.0.5 throughout) [35], based on Hamming genetic distance. A cut-off to define duplicates was determined by finding the maximum genetic distance between any known pair of either technical or biological duplicates. Any pair of samples with a distance below this level, identified using an R script, were designated as duplicates, and groups of the same clone identified. One sample of each group of identical samples, was selected to represent the group. A unique set of landraces was thus defined. All R scripts are provided in S3 File.

Initially a discriminant analysis of principal components (DAPC), an assumption-free multivariate clustering method [36] was conducted in R to assess the genetic relationship among the unique landraces by the seven Zones (S3 File). Genetic relationships were calculated based on Hamming distance and illustrated in a circular dendrogram using the ggtree package (ver. 2.4.1) in R. In addition, a minimum spanning network (MSN) was used to visualize population structure using the poppr (ver. 2.9.1) R Package [37] (S3 File). This clusters multilocus genotypes (MLG) by the genetic distances between them. Each MLG is a node, which is connected by the minimum distance between samples, represented by the edges. This allows for reticulations i.e. nodes with identical genetic distances can be connected in a network, as opposed to a tree whereby pairwise nodes are only connected to one other node with the shortest distances, even if several nodes share the same minimum genetic distance.

Genetic diversity was assessed by Zones using Shannon’s Index [38] and the package hierfstat (ver. 0.5-7) [39] in R (S3 File). Nei’s genetic distance was calculated among Zones, and the relationships displayed using the unweighted neighbour-joining method in Darwin v5.0 [40].

#### Virus elimination

Cassava stakes of the unique set of landraces were collected from the field genebank at Naliendele and taken to the regional plant health quarantine station at Kenya Plant Health Inspectorate Service (KEPHIS), Muguga, Kenya, for virus elimination and subsequent conservation at a designated facility. Thermotherapy followed by meristem tip culture was used for virus elimination according to the manual by Ferguson *et al*. [41].

## Results

A total of 193 farmers growing cassava landraces were identified and interviewed (S4 File) and 427 landraces were collected from their fields and homesteads, across seven zones of Tanzania, namely Western Zone, Lake Zone, Northern Zone, Central Zone, Coastal Zone, Zanzibar Zone and the Southern Highlands Zone (Fig 1).The number of sites where interviews and sampling was conducted ranged from 14 in the Western Zone to 42 in the Southern Highlands, with the number of samples taken ranging from 30 in the Western Zone to 81 in the Southern Highlands (Table 1). Two sites were sampled in the Southern Zone but were included within the Southern Highlands for analysis purposes.

**Table 1:** Number of collection sites and landrace samples collected per geographical zone.

Zone	Collector number prefix	Number of collection sites	Number of landraces samples collected
Central Zone	EPM; KSF (93-103)	35	79
Coastal Zone	KS (01-42; 65-75)	27	53
Lake Zone	KSF (1-62)	18	62
Northern Zone	Horti; KS (43-64)	27	69
Southern Highlands	BKP	42	81
Western Zone	KSF (63-92)	14	30
Zanzibar	SMS	30	53
Total		193	427

### Respondent profiles and their farms

Women made up 26% of the interviewed farmers while men were 74% with no attempt to meet a predefined gender ratio. Their ages ranged from 18 to 86 years and the majority (91%) were the principal caretakers/decision makers with regard to cassava cultivation in their respective households, meaning they were the people who decided which landraces or improved varieties and how many of each cultivar would be grown. All data from key informant interviews is provided in S5 File with landrace collection data in S6 File. In terms of the estimated number of years the individual farmers had been cultivating cassava, 25% had less than 5 years, 31% had between 5-19 years, 40% had between 20-49 years and only 2% had 50 years or more of growing cassava; 2% did not state their years of cassava growing. The majority (66%) of the interviewed farmers had at least some primary level of education with 10% having secondary education and 2% tertiary education. The remainder had no education. Eighty percent of key informants relied on farming as their main occupation and 80% as their main source of income. Others were engaged in formal or informal wage work or business.

Thirteen and a half percent of farmers had access to less than 1 acre of agricultural land, 42.7% had access to between 1-4.9 acres, 24.5% had access to 5-9.9 acres, 17.7% had access to more than 10 acres of agricultural land and 1% did not state the size of agricultural land they have access to. All the farmers who had access to less than 1 acre of agricultural land grew cassava on all their land. Of the 82 farmers who made up the 42.7% of farmers who had access to between 1-4.9 acres of land, 51 (62.2%) of them had cassava grown on all the available land. As the size of the individual farms increased from 5 acres to 10 acres or more, the area of land allocated for cassava decreased; more than half of those that had access to 5-9.9 acres of agricultural land had cassava cultivated on land between less than 1 acre to 4.9 acres.

### The landraces

The 427 landraces collected from the farmers had mostly local names. Some of the names, when translated to English, described characteristics of the landraces, for example ‘too sweet to eat’, ‘hunger fighter’, ‘needle leaves’, ‘sit and eat’ and ‘the great’. The varieties were named by various communities based on their morphological or agronomic traits or after the person who introduced them or the place where they originated.

**Fig 2:**
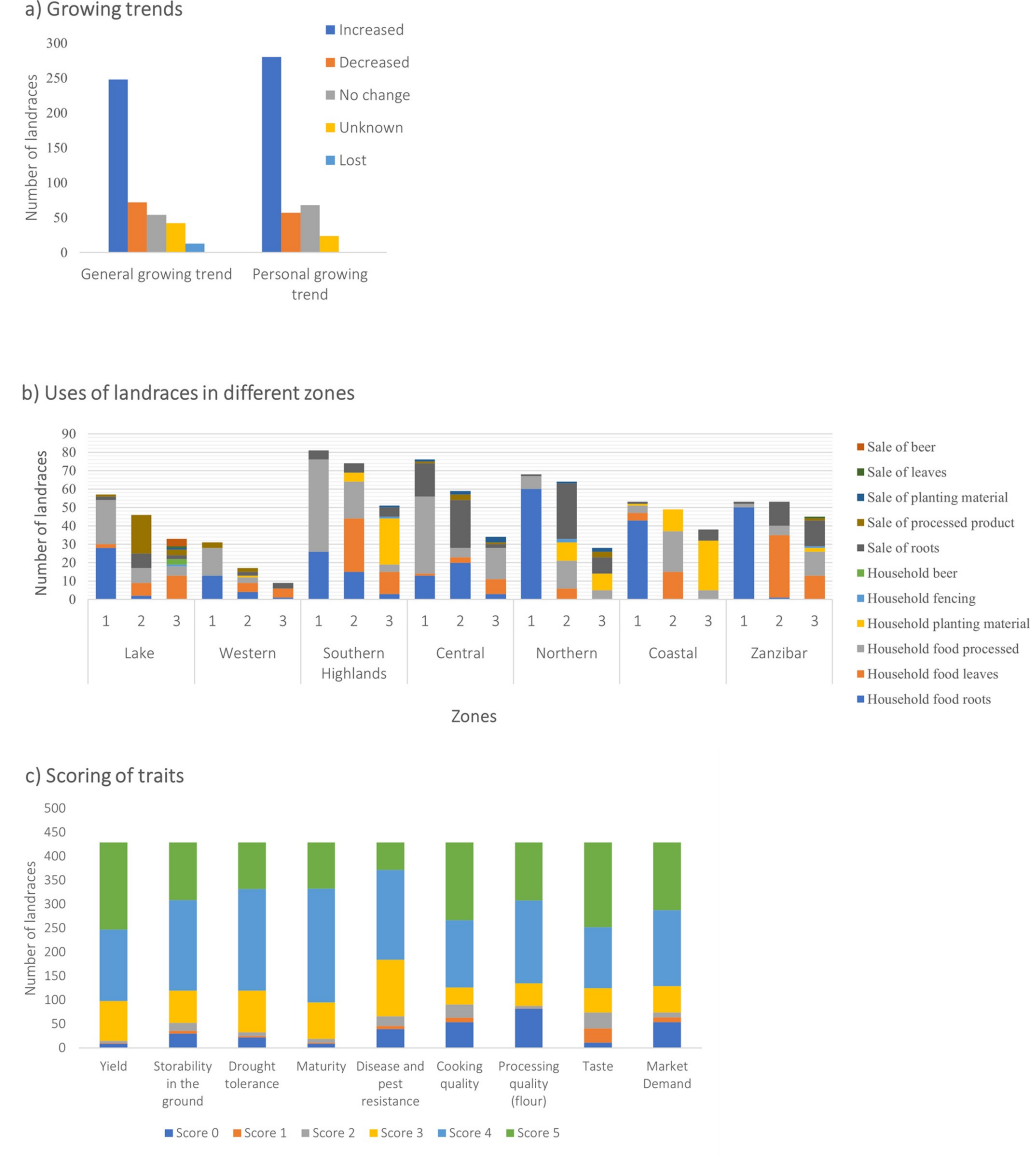
Tanzanian landraces show different growing trends, uses and trait scores: (a) General and personal growing trends of individual landraces; (b) Uses of landraces as first (1), second (2) and third (3) priority in different zones. ‘Household food roots’ equates to fresh consumption (raw, boiled or fried), whereas ‘household food processed’, means consumed as a flour; (c) Trait scores for landraces as rated by farmers where 0 = don’t know, 1 = very bad, 2 = bad, 3 = fair, 4 = good and 5 = very good. Data supporting Fig 2B, is provided S7 File.

Most of the landraces (87.9%) were best estimated by the farmers to have arrived in their communities from a time period ranging from 1950s or before, to the 2010s, the remaining 12.1% the farmers had no idea when they arrived in their communities. In the preceding five years, the majority of the landraces had seen an increase in their general cultivation in the area where they were collected and by the individual farmers from whom they were collected (Fig 2A) meaning that many of the landraces have gained popularity in the past five years. Fewer landraces had a decreased or stagnant growing trend, while for others, the trend was unknown. The main reasons given for increased cultivation of particular landraces in order of rank were high yield (32%) and food security (32%), high marketability (22%), good taste (17%), early maturity (7%), disease and pest resistance (6%), short cooking (boiling) time and cooking quality (6%). The most frequent reasons for reducing cultivation of particular landraces included susceptibility of pests and diseases (27%), low yield (27%), unmarketable (17%), late maturity (10%), bitter taste (10%) and availability of better varieties (10%).

Based on the categorial data collected on the use of cassava it was clear that growing cassava for use as household food (boiled fresh roots or dried and milled into a flour for a variety of culinary uses) and for consumption of leaves are common uses in all regions (Fig 2B). In the Lake (53%), Northern (90%), Coastal (91%) and Zanzibar Zones (96%) a larger proportion of the landraces were consumed as fresh roots (figures in parentheses refer to the proportion of respondents that cited the primary use of cassava as fresh roots as opposed to processed into flour); while in the Southern Highlands (66%), Western (54%) and Central (76%) Zones a greater proportion of the collected landraces were grown primarily for processing into flour for home consumption (the figures in parentheses refer to the proportion of respondents that cited the primary use of cassava as processed for flour as opposed to fresh consumption) (Fig 2B). This suggests that a larger proportion of landraces from the Southern Highlands, Western and Central Zones are bitter cultivars. The sweetness or bitterness of cassava cultivars is associated with cyanogenic glycoside concentrations. Sweet cultivars have low amounts of cyanogenic glycosides and can be safely consumed with simple boiling or even raw, whereas bitter cultivars have high concentrations of cyanogenic glycosides (>50ppm fresh weight [16]) and require detoxification and processing before consumption in the form of flour or similar form.

Landraces were categorized according to their uses. If it was mentioned that either the primary, secondary or tertiary use included both fresh consumption and processing into flour, the landrace was classified as ‘dual purpose’; if fresh consumption was mentioned only, the landrace was classified as ‘fresh’ and if processing was mentioned alone, it was classified as ‘flour production’. The above classification may not accurately reflect the physical attributes of the landrace as use is influenced by cultural practices; for example, the fact that in the coastal zone, landraces are only used for fresh consumption, does not mean that the landraces could not be used for flour production. It does imply that they are sweet enough for fresh consumption.

Based on the use, cited by key informants, 164 (38%) landraces were used purely for fresh consumption, 145 (34%) were dual purpose and 103 (24%) were for processing (flour) only. and 15 (4%) had unknown uses (S5 File and S8.1 Fig in S8 File).

The farmers were asked to rate various qualities of their landraces on a scale of 1 to 5 (1 being very bad and 5 being very good), a score of 0 was given if the informant couldn’t comment, for instance if they hadn’t yet experienced a drought with this landrace or didn’t participate in the market. Fig 2C shows the distribution of the quality scores for various traits.

The distribution of scores for the storability of the landrace in the ground after maturity indicates that the majority of the landraces store fairly well to very good. Farmers who scored 5 for storability (8.3% of landraces), indicated that the landraces could stay in the ground for 5 years or more after maturity. Those that scored 1 or 2 (5.5% of landraces) could be stored for less than 18 months which was considered poor. This was also associated with susceptibility to CBSD root necrosis where root necrosis increases dramatically after six months in susceptible varieties.

There appeared to be great discrepancy, even understanding, of what constituted early, medium or late maturing varieties. If cassava was being used for income generation or food, a short time to maturity was preferred (< 12 months), however if the crop was being used purely for food security, a late maturing variety (> 12 months) which could be stored for more than 5 years was preferred. Drought tolerance scores indicated that 95% of farmers had experienced this stress but 97.3% of landraces still performed fairly well to very good (scores 3-5) under such conditions, in fact farmers commented that the yield was unchanged in drought years in 13 landraces.

Pest and disease scores and comments for many of the landraces indicated that more than half of the landraces had good to very good resistance to pest and diseases, although many of the farmers could not recognize pests or diseases.

Two hundred and ninety-two (97%) of the 300 landraces that scored 4 or 5 for cooking quality also scored 4 or 5 for taste indicating they were ‘sweet’. These were good for consuming raw, boiled or fried, and made up 71% of all 427 landraces. This is similar to the estimate of 72% of fresh (38%) and dual purpose (34%) calculated according to categorization of landraces based on uses provided by key informant interviews discussed above. The distribution of cooking quality scores according to purpose or use is given in S8.2 Fig in S8 File and taste scores with purpose or use in S8.3 Fig in S8 File. Those scoring 4 or 5 for cooking quality were regarded as being ‘mealy’. Mealy roots are friable, they cook or boil easily and become soft and floury in texture. However, six landraces that scored 4 were regarded as good in terms of softness but they took a longer time to boil, one was also regarded as having good cooking quality but needed soaking first while another had good cooking quality but deteriorated during the rainy season. Comments on 162 landraces that scored 5 for cooking quality referred to the fact that they got soft after a short time of boiling and some were also listed as having high starch or dry matter content. The correlation between cooking quality and taste across all landraces was reasonably high, R=0.8169 (S8.1 Table in S8 File) and R^2^=0.6425 (S8.2 Table in S8 File). Thirty-six (8.4%) scored 1 or 2 for cooking quality as they were predominantly used for flour (ugali) making. Of these, only three scored 4 or 5 for taste, with the remaining 33 scoring 1-3 indicating they were largely bitter varieties. All of these varieties scored 4 or 5 for processing quality (S8.5 Fig in S8 File). Fifty-three (12%) of the landraces scored 1 to 3 for processing quality indicating they were not good for flour making. All but two of these varieties scored above 3 for taste and cooking quality, indicating they were sweet and were predominantly for fresh consumption. Responses relating to an additional 43 (10%) landraces indicated that the respondents had never tried to process the roots into flour (S8.5 Fig in S8 File).

Scores indicate that the market demand of a number of landraces is quite high. Seventy-four (17%) landraces that scored 1 to 3 had poor market demand and of these 47% scored 1 to 3 for taste, indicating they were bitter. Two hundred and ninety-eight (70%) landraces scored 4 or 5 in terms of market demand and of these 228 (77%) landraces scored 4 or 5 in terms of taste indicating they were reasonably sweet cultivars. Indeed, in terms of market demand the highest correlation of scores was with cooking quality (R=0.4028), followed by taste (R=0.3496) and yield (R=0.3401) (S8.1 and S8.2 Table in S8 File). Correlations between cooking quality, taste, processing quality, yield and market demand are provided in S8.1 and S8.2 Table in S8 File.

According to data from the key informant interviews, 62% of farmers across all zones in Tanzania grow cassava in multiple plots as a primary means of conservation and maintaining the landrace. This conservation method is coupled with the practice of giving out the landraces to others to plant. In addition, farmers also stagger harvesting and re-planting to spread risk, so they do not harvest the entire crop at once.

Sixty-three percent of farmers said that in the past five years they had grown the landraces from their own planting material, with 13% supplementing their own planting material with planting material that they had been given from either a person in their community or outside their community. Only one supplemented their own planting material by purchasing planting material. Twenty percent of farmers relied on gifts of planting material alone. Eighty-three percent of farmers had given cassava stakes of their landraces as gifts and only 7.7% had sold stakes.

#### Assessment of cooking quality and organoleptic traits

The list of 22 clones selected using information on cooking quality traits and organoleptic traits collected during the key informant interviews, and results from the on-station panel assessment, together with the laboratory analyses are given in S9 File. Storage roots from 16 of the 22 clones selected were analysed for CNP content (mg/kg) and softness in the laboratory. Some landraces such as BKP13 (synonymous with EPM24, BKP34, BKP04, BKP09 and EPM27) collected in the Southern Highland Zone (BKP) and the Central Zone (EPM) were clearly used specifically for flour production. This was bitter according to the key informant interviews undertaken during collection, and in the on-station panel assessment. BKP13 also had the highest level of CNP at 175mg/kg fresh weight. It was also amongst the hardest post-boiling according to the on-station toothpick test and the panel assessment. It was also the hardest according to the laboratory probe tests and had the lowest score for mealiness. Iragaba *et al*., [42] found a strong agreement between ordinal scores of root softness from consumer testing and penetrometer measurements of cooked roots.

BKP29, a unique landrace collected in the Southern Highland Zone, had the lowest score for CNP (29mg/kg fresh weight) and was classified as sweet both from key informant interviews and on-station panel assessments. It was also softest according to laboratory probe tests, but oddly had scores indicating hardness by the panel members. The panel did indicate that it was extremely mealy. This was clearly a fresh consumption variety with poor flour making characteristics.

HORTI_23 was collected 11 times, from the Southern Highland Zone (BKP63), the Coastal Zone (KS38, KS66) and the Northern Zone (HORTI_12, HORTI_21, HORTI_14, HORTI_43, HORTI_19, KS45, KS50) and had the shortest time to cook (boil) (13 min). Likewise, KS70 (same as KS73) from the Coastal Zone, also cooked in 13min. Both of these landraces were scored as sweet, extremely mealy and good or very good for both boiling and processing into flour. SMS11 from Zanzibar, which was not collected elsewhere, had the longest cooking time of 36min. It was also amongst the hardest according to laboratory probe tests and had a low mealiness score according to the panel assessment. It was clearly used for fresh consumption as the processing quality was unknown.

#### Genotyping results

Good quality DNA was obtained from 406 of the 427 landraces that were collected as some failed to sprout in the field genebank. Four landraces were excluded for having >5.5% missing SNP data, and KSF61 was deleted as it matched with the breeding line TME204, leaving 401 landraces, and a total dataset of 490 entries including landrace and breeding references and biological and technical replicates (see Table 2). An initial dataset of 36,153 SNPs was generated, however after deleting all SNPs with greater than 5% missing data and all monomorphic loci, the set was reduced to 20,072 SNPs.

**Table 2:** Outcome from the identification of unique landraces based on molecular analysis.

Category/Zone	Number of entries in analysis	Number of landraces collected more than once, in the same or a different Zone	Number of landraces collected only once	Number of unique landraces per Zone*	Number of landraces shared across Zones	Number of landraces shared across two or three zones	% landraces that occur in two or more regions	Number of biological and technical replicates
Landrace reference	35							23
Breeding line	21							5
Southern Highland (SH)	79	16	41	57	2(CeZ), 1(NZ), 1(NZ & Co), 1(Ce &WZ)	5	8.8	
Central (CeZ)	57	20	25	45	2(SH), 1(WZ), 1(LZ), 1(SH & WZ), 1(NZ & WZ), 1(LZ &WZ)	7	15.6	2
Northern (NZ)	65	16	26	42	1(SH), 1(CoZ & SH), 3(CoZ), 1(CeZ &WZ), 2(ZZ)	8	19	1
Coastal (CoZ)	53	17	22	39	3(ZZ), 1(NZ &SH), 3(NZ)	7	17.9	
Lake (LZ)	58	9	37	46	1(CeZ & WZ), 2(WZ), 1(CeZ)	4	8.7	
Western (WZ)	37	6	24	30	2(LZ), 1(CeZ), 1(CeZ & LZ), 1(CeZ & NZ), 1(CeZ &SH)	6	16.7	
Zanzibar (ZZ)	53	13	28	41	3(CoZ), 2(NZ)	5	12.2	1
Total	458	97	203	300				32

* If a landrace is found in two or more zones, it is counted once in each zone, so the total number of unique clones collected appears more than the actual 277 unique clone.

The raw dataset and distance matrix can be found in S10 and S11 Files. The average distance between biological replicates was 0.00824 and the maximum distance was 0.02112 between two Nachinyaya lines, followed by 0.01656, again, between two Nachinyaya lines. The average distance between technical replicates was 0.01245, higher than the biological replicates, with a maximum distance of 0.02027 which was similar to the biological replicates. A cutoff of less 0.022 was used to define a duplicate, and this is illustrated in a dendrogram in S12 File. The residual distance between technical duplicates is most likely due to miss-calling of heterozygotes as homozygotes from low sequencing read-depth, as is typical in high-multiplexing, sequence-based genotyping methods [43]. A total of 285 unique landraces were identified including landrace references and 277 unique landraces excluding landrace references (S13 File). Almost all the duplicates had different names based on the key informant interviews.

In total 37 landraces could be identified through similarity with a reference landrace. Seventeen of the 35 reference landraces matched with at least one collected landrace thus aiding with identification and eliminating duplicates. All reference landraces that found a match were from Tanzania, except Mbundumali which is thought to be from Malawi, but was collected in the Southern Highlands of Tanzania, adjacent to Malawi (BKP10, 35, 37 and 60) as well as in the Central Zone (EPM23). Two of the reference landraces, Namikonga (Tanzania) and XinoNn’gole (Mozambique) were found to be identical. In total 285 unique landraces were identified, including those matching with the 17 Tanzanian landrace references (including Mbundumali) (S13 File). The reference landrace Aipin Valenca was found in the Northern Zone only, Mreteta was found in the Southern Highlands only, Dodoma, Mumba, Hombolo, Makutupora, Kachanga and Songambele were found in the arid Central Zone only, Namikonga in the Northern Zone only, Kiroba and Mfaransa in the Coastal Zone only, Kizimbani in Zanzibar only, and Lyongo Kwimba in the Lake Zone (S13 File).

The maximum number of landraces were collected in the Southern Highlands (57) followed by the Lake Zone (46) and closely followed by the Central Zone (45). Thirty landraces were collected in the smaller Western Zone. Seventy-eight landraces of the 277 unique landraces (28%), were collected more than once, either in the same zone, or a different zone. Only four landraces were collected in a maximum of three zones. None of the landraces was collected across more than three of the seven Zones (Table 2). The number of unique landraces collected per zone, the number of landraces collected only once, and the number of landraces that were common across two or more zones is provided in Table 2.

Interestingly, the island of Pemba in the Zanzibar Zone, only shared one of its 16 landraces with Zanzibar, with the remainder being unique to the island. The Northern Zone shared the maximum percentage of landraces with other zones (19%), followed by the Coastal Zone (17.9%) with Zanzibar, the Northern Zone or the Southern Highlands Zone (Table 2). The Lake Zone only shared 4 (9%) landraces with the Western Zone and one with the Central Zone (Table 2).

**Fig 3:**
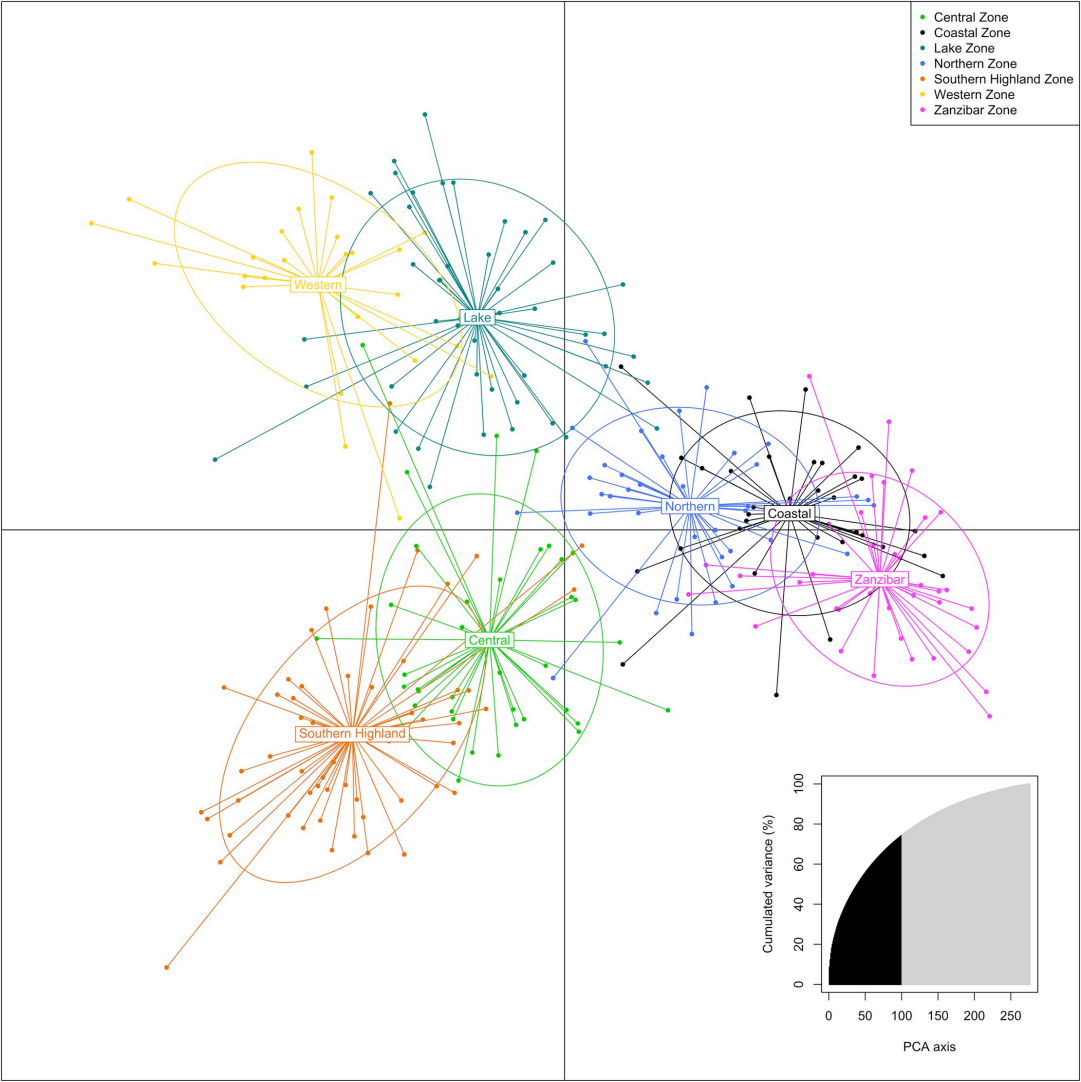
DAPC plot showing the relationship of cassava landraces from seven zones of Tanzania. The number of axes retained in the Principal Component Analysis (PCA) were 100 which account for 74.2% of the variation (inset).

**Fig 4:**
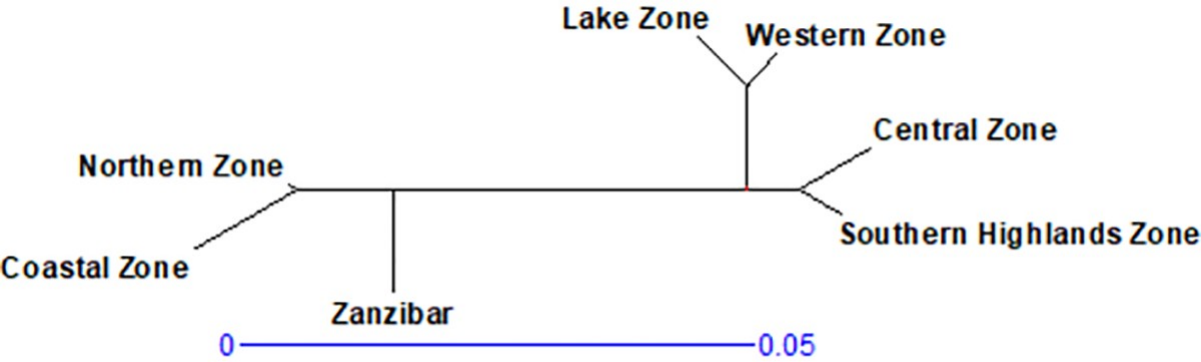
Relationship of geographical zones based on the genetic distance of 401 cassava landraces collected therein. Nei’s genetic distance was calculated among Zones, and the relationships displayed using the unweighted neighbour-joining method in Darwin v5.0 [40].

A DAPC plot of the relationship between 401 landraces from the seven zones is given in Fig 3. The number of axes retained in the analysis were 100 which account for 74.2% of the variation. A circular dendrogram is provided in S14 File with an MSN in S15 File. Genetic relationships among seven Zones, based on Nei’s genetic distance is given in Fig 4. The greatest differentiation between Zones, according to Fst statistics, was between the Coastal Zone and the Western Zone (0.07) and the least differentiation between the Central Zone and the Southern Highlands Zone (0.013). Based on 277 unique landraces and according to Shannon’s Index, the Southern Highlands Zone had the greatest diversity (4.007), and the Western Zone the least (3.219). Interestingly, admixture analysis, which attributes hypothetical ancestral proportions, did not give meaningful results even with duplicates removed, possibly because the genotypes were too closely related to one another.

Representatives of 158 unique clones were sent to KEPHIS, Kenya, for virus elimination in preparation for conservation. Of these 82 are currently virus free, 34 are still in the virus elimination pipeline and 42 either did not establish at KEPHIS or were lost during the virus elimination process. This process is on-going.

## Discussion

Cassava landraces have provided vital food security and income in Tanzania for centuries. They have adapted over the years to prevailing agro-ecological conditions and have been selected by farmers based on a range of criteria, ranging from food security, preferred cooking and eating quality traits and market demand [44]. The existence of these landraces is now threatened not only by virus diseases (CBSD and CMD), but by the influx of a few disease-resistant, higher yielding improved cultivars [44]. This is likely to reduce the diversity of cassava grown in Tanzania. Unfortunately, cassava landraces from East, South and Central Africa are under-represented in global germplasm repositories yet offer important diversity [3]. Here we describe the collection of Tanzanian cassava landraces and associated farmer knowledge, as we strive to conserve in perpetuity as many of these landraces as possible. We also describe diversity at the molecular level and investigate the cooking and eating qualities of selected landraces.

Interestingly 74% of the farmers interviewed, who were actively involved in decision making as to which cultivars to grow, and how much of each cultivar to grow, were male. Mtunguja *et al*., [44] also found that the selection of cassava landraces in Tanzania was done by both men and women. Farming was the main form of income for the majority of these people. Over 40% of farmers had 1 – 4.9 acres and the majority of farmers grew cassava on all of their land. This demonstrates the importance of cassava in Tanzania. The vast majority of farmers grew more than one variety of cassava to take care of diverse needs and spread risk. This is typical of subsistence farming systems.

Like others have found, preferences for cooking and eating qualities were very much depended on the intended use [45–48]. There were clearly two main ways of preparing cassava for consumption, either fresh, normally by peeling, chopping, and boiling or frying; or by peeling, chopping, drying and pounding or milling into a flour for various uses. Unlike in West Africa, no fermentation is involved. Use as flour was more common in the Southern Highlands, Western and Central Zones, as opposed to the Lake, Northern, Coastal and Zanzibar Zones where fresh consumption is more common. These different uses reflect the importance of various traits in the cassava storage roots.

Key informant interviews and on-station and laboratory assessments indicate that those used for flour were generally bitter with relatively higher CNP levels (up to 175mg/kg fresh weight). It also appeared that in these cultivars softness after boiling was not a priority, nor was mealiness. An example of such a variety was BKP13 (with five synonyms from the Southern Highlands and Central Zones) which could be incorporated into breeding programs to confer both agro-ecological adaptation as well as other processing characteristics important to this product profile. In the neighbouring region of northern Malawi where cassava is consumed as a staple crop, Benesi *et al*. [45] found that very bitter cultivars were preferred.

Landraces used more specifically for fresh consumption were characterized by a short time to cook (minimum 13min), became soft after boiling, and mealy. Clearly, the texture of cassava products is a critical factor in the acceptance of fresh cassava [27]. Mealiness is correlated with dry matter content and thought to be influenced by pectin content [27,49–51]. A unique variety, BKP29, again from the Southern Highlands Zone, is an example of such a variety.

According to information on use from key informant interviews, 34% of landraces were dual purpose, being used for flour, but also for fresh consumption, 38% were used purely for fresh consumption (although this does not imply that their qualities were not suitable for flour production, as in several Zones, flour making is not traditional), and 24% were used for processing only. Based on these figures, 72% of landraces were consumed fresh. This was similar to the estimate of 71% based on scores of 4 and 5 for cooking quality and taste, although from these scores, and scores of processing quality, it was estimated that 45% were dual purpose. This highlights three product profiles; flour production, fresh consumption and dual purpose.

Genetic distance based on SNP fingerprinting was used to identify duplicate samples among the collected landraces and to visualize genetic relationships among both landraces and regions. A similar approach was used by Rabbi *et al*. [52] to assess rates of improved variety adoption. SNP markers have been used previously to distinguish morphologically similar cassava landraces [53]. Interestingly, Oyesigye *et al*. [54] found that XinoNn’gole from Mozambique was genetically identical to Namikonga, a CBSD resistant variety, based on a small number of SNPs. This is confirmed here using high density SNP genotyping. This identified a set of 277 unique landraces, although it is possible that some of these may be improved cultivars that were not identified by the breeders on the mission and did not match any of the reference breeding lines included in the genotyping study. The fact that almost all of the duplicates within sets had different names according to the key informant interview, was expected, and has been found by others [52].

Interestingly, most of the individual landraces were not widely distributed across the country. Only 28% of landraces were collected more than once, and only four landraces were collected in a maximum of three zones. From the key informant interviews, 35% of the landraces were acquired either as a gift or purchase within the community, sometimes supplementing their own seed (usually stem cuttings from the preceding crop). This has been documented previously by Mtunguja *et al*. [44] who found the flow of seed within and outside the village, but little introduction of new cultivars. In the absence of a formal seed system, it appears that the spread of the majority of cultivars between communities is relatively limited, and even more limited between zones. This explains to some extent the challenges faced in varietal dissemination [55], that may rely on diffusion between farmers. Data here indicate that this is not as widespread as perhaps initially thought.

This study found that a large number of unique landraces are grown in farmers’ fields. A similar situation was found in cassava by Kizito *et al*. [56], Benesi *et al*. [45] and Mtunguja *et al*. [53]. This is somewhat surprising in an apparently predominantly clonally propagated crop; however, soil seed banks are an essential feature of the ecology of landrace populations of cassava [9]. Cassava produces largely cross-pollinated seed in dehiscent seed capsules which shatter dispersing seed onto the soil. Farmers incorporate the resulting seedlings to supplement their planted cultivars. This mixed clonal/sexual reproductive system tends to increase diversity [9]. This diversity constitutes an important element for the livelihood strategies of these farmers.

There was a clear structure in the diversity (Figs 3 and 4) with the Coastal, Northern and Zanzibar Zones clustered together, and separated from all other Zones. Within the remaining Zones, the Central and Southern Highlands Zone were more similar to each other, and the Lake Zone and Western Zone. This pattern may reflect the two distinct avenues for introduction of cassava germplasm to the country; from the coast, via the islands of Pemba and Zanzibar, and overland from the West via Congo and Angola. It may also reflect adaptation to the agro-ecology of these areas as well as cooking and organoleptic preferences, defined by how cassava is consumed, either fresh consumption which is common in coastal areas or processed into a flour which is more popular in southern, central and western regions. Elias *et al*. [57] and Clement *et al*. [16] recognize a post-domestication geographical separation of cassava into sweet and bitter groups in the Amazonian region, based on root cyanide content. Although sweet cassava is generally cultivated throughout the Neotropics, it dominates in the western and southern regions of the Amazon river basin, while the north-eastern portion is dominated by bitter cassava [58–60] which is predominantly used for starch extraction and cassava flour production. It is possible that the structure that is seen in Tanzanian cassava landrace diversity may be influenced by the prominence of sweet or bitter cultivars.

Little difference in levels of diversity were observed, with the Southern Highlands exhibiting slightly more diversity. Benesi *et al*. [45] found that cultivars from the northern Malawi, neighbouring Tanzania, were more diverse than those from the central and southern regions.

## Conclusions

Cassava is clearly a vital crop for smallholder farmers in Tanzania and a very large number of different landraces are grown. This likely reflects the incorporation by farmers of seedlings from the soil seed bank. The observed genetic relationships among zones are likely due to independent introductions of cassava into the country, adaptation to prevailing agro-ecological conditions and farmer selections according to the intended use or market demands. Several product profiles are evident, including fresh consumption, flour production, dual purpose and longer-term food security. Each of these products have different trait requirements. It is important that the consolidated adaptation to all these specific elements accumulated in landraces are conserved and utilized to improve customer acceptance and adoption of new cultivars. The majority of individual landraces were not widely distributed across the country with limited farmer-to-farmer diffusion with implications for seed systems.

## Supporting information

S1 File List of the reference landraces, biological and technical replicates used to identify unknown landraces and define a cut-off to specify a unique landrace. (XLSX)

S2 File List of reference breeding lines and information on technical replicates. (XLSX)

S3 File R Scripts for analysis of genotypic data. (TXT)

S4 File Geographical location of sampling sites and key informant profiles. (XLSX)

S5 File Data from key informant assessment of cassava landraces. (XLSX)

S6 File Data of cassava landrace morphological characteristics. (XLSX)

S7 File Primary, secondary and tertiary uses of landraces according to Zone. This data forms the basis of Fig 2B.
(XLSX)

S8 File Proportion of landraces used for different purposes, score distributions for cooking quality, taste and processing quality, and correlation coefficients between taste, cooking quality, processing quality, market demand and yield. (PDF)

S9 File On-station measurement of cooking quality and organoleptic traits in comparison to information obtained during key informant interviews with farmers, of 22 farmer-preferred varieties in Tanzania. (XLSX)

S10 File SNP input file_490 individuals. (CSV)

S11 File Distance matrix_490 individuals. (CSV)

S12 File Dendrogram to assist in the identification of duplicates. (PDF)

S13 File Duplicates, unique landraces and matches with known landraces, including those available *in vitro*. (XLSX)

S14 File Circular dendrogram of 401 landraces collected in Tanzania, coded according to the Zone in which they were collected. (PDF)

S15 File Minimum spanning network of 401 landraces collected in Tanzania, coded according to the Zone in which they were collected. (PDF)
