# Supplementary material for: Collection, genotyping and virus elimination of cassava landraces from Tanzania and documentation of farmer knowledge
Source: PLoS One. 2021 Aug 17;16(8):e0255326. doi: 10.1371/journal.pone.0255326 (PMC8370617; doi:10.1371/journal.pone.0255326)
Supplement: S8 File — (PDF) [file pone.0255326.s008.pdf]

PONE-D-20-24321

Ferguson et al. Collection, Genotyping and Virus Elimination of Cassava Landraces from Tanzania and Documentation of Farmer Knowledge. PloS One.

Supplementary file S8:

Fig. S8.1. Number of landraces used for fresh consumption alone, dual purpose (fresh consumption and flour production) and flour production alone based on 412 landraces for which data was available.

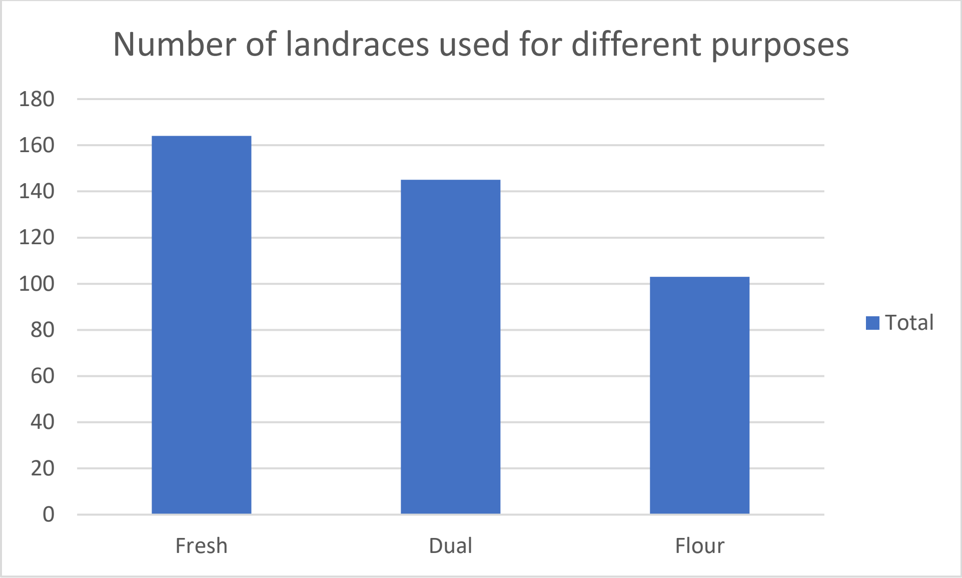

Fig. S8.2. Score distribution for cooking quality according to use, based on 412 landraces, where 1 is very poor and 5 is excellent. There were many unknown scores for landraces used for flour production as they are not cooked fresh.

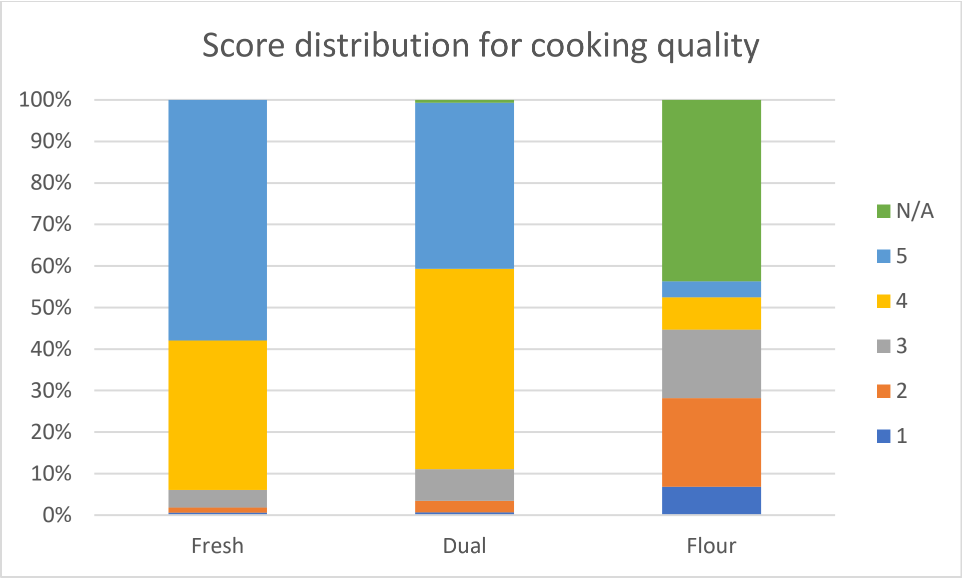

Fig. S8.3. Score distribution for taste according to use, based on 412 landraces, where 1 is very poor and 5 is excellent.

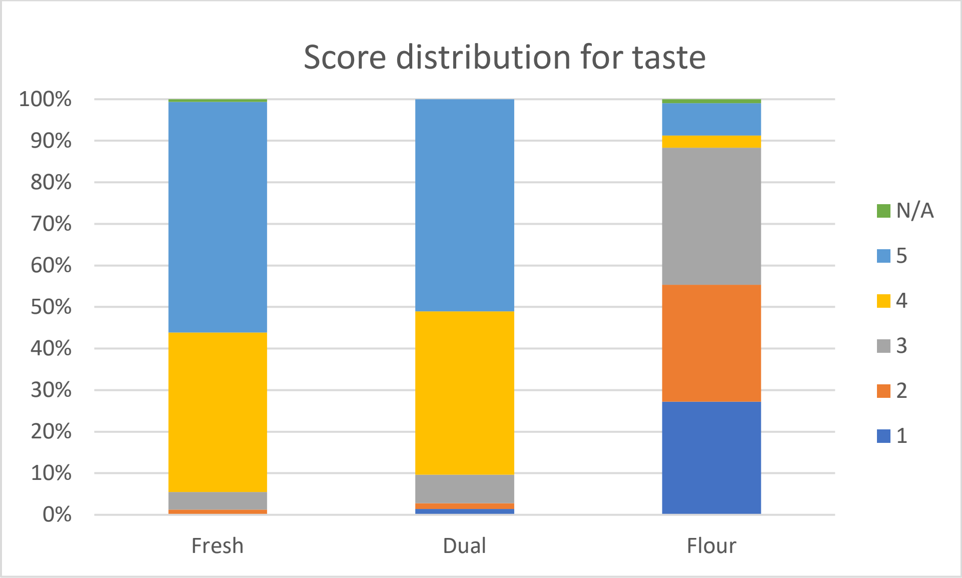

Fig. S8.4. Score distribution for processing quality according to use, based on 412 landraces, where 1 is very poor and 5 is excellent. There were many unknown scores for landraces used for flour production as they are not cooked fresh.

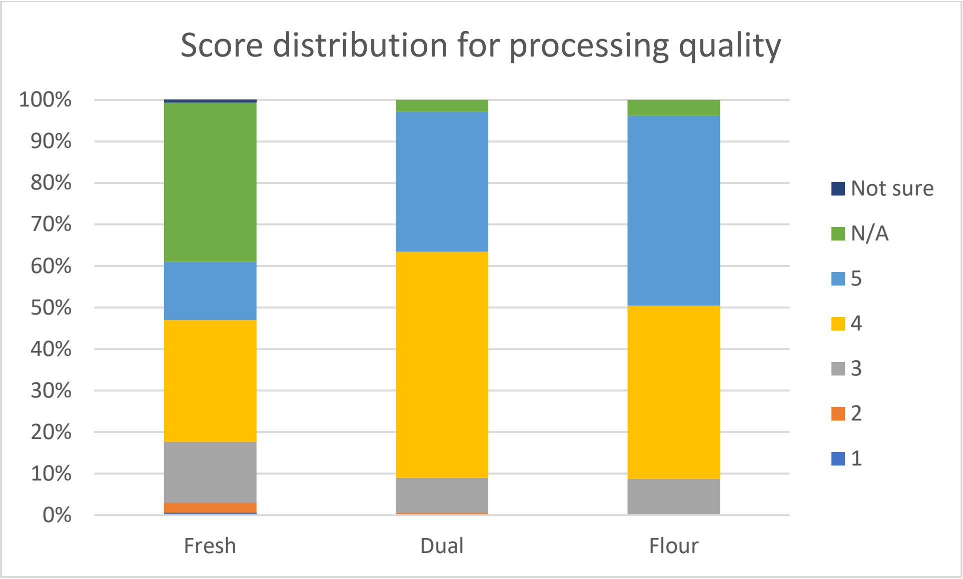

Table 8.1. Correlation coefficient (R), explaining the direction and strength of the linear association amongst the variables yield, market demand, processing quality, cooking quality and taste

	Yield	Market demand	Taste	Cooking quality	Processing quality
Yield	0				
Market demand	0.3401	0			
Taste	0.1988	0.3496	0		
Cooking quality	0.2835	0.4028	0.8169	0	
Processing quality	0.2022	0.2267	-0.0267	0.0845	0

Table 8.2. Correlation (R^2), explaining the extent to which the variance of one variable explains the variance of another, among yield, market demand, processing quality, cooking quality and taste.

	Yield	Market demand	Taste	Cooking quality	Processing quality
Yield	0				
Market demand	0.1157	0			
Taste	0.0395	0.1222	0		
Cooking quality	0.0804	0.1622	0.6425	0	
Processing quality	0.0409	0.0514	0.0007	0.0071	0
