# Supplementary material for: Collection, genotyping and virus elimination of cassava landraces from Tanzania and documentation of farmer knowledge
Source: PLoS One. 2021 Aug 17;16(8):e0255326. doi: 10.1371/journal.pone.0255326 (PMC8370617; doi:10.1371/journal.pone.0255326)

PONE-D-20-24321

Ferguson et al. Collection, Genotyping and Virus Elimination of Cassava Landraces from Tanzania and Documentation of Farmer Knowledge. Plos One

Supplementary file_S14: Circular dendrogram of 401 landraces collected in Tanzania, coded according to the Zone where they were collected.

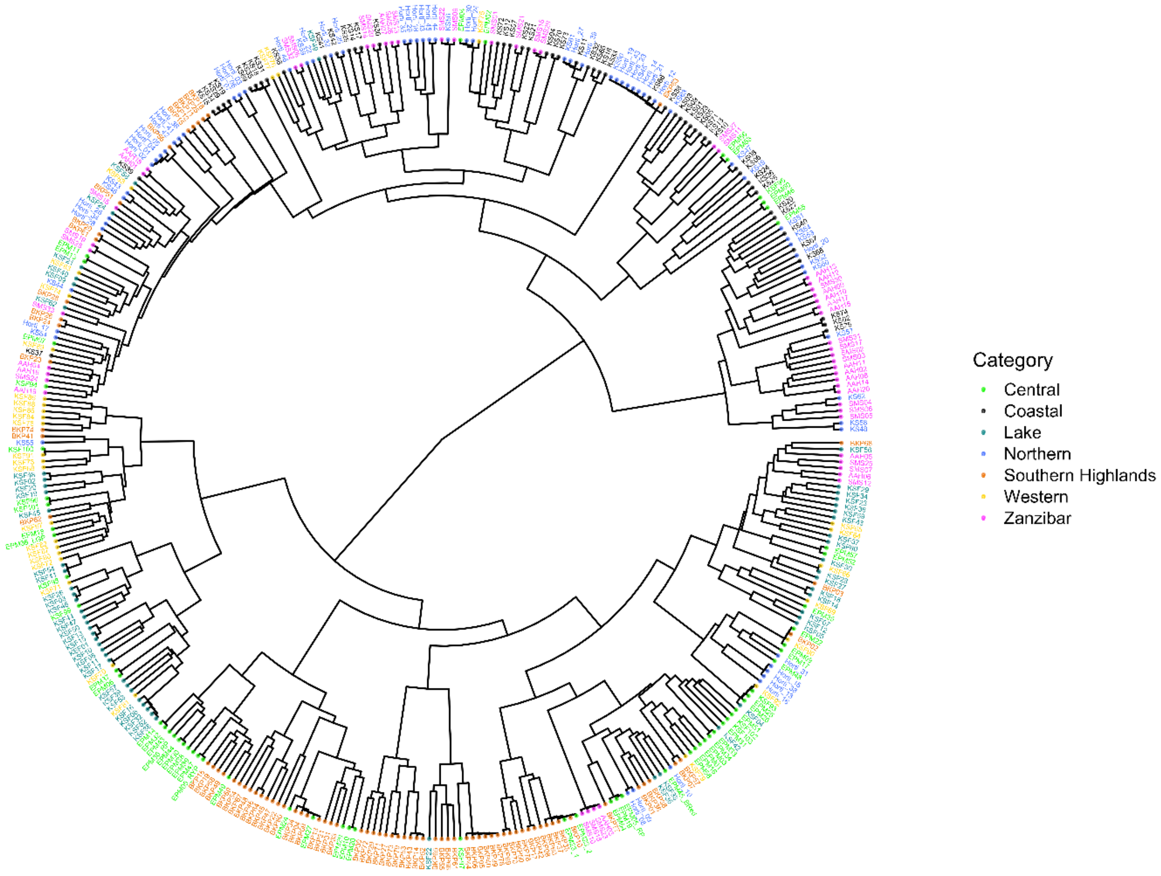

Supplement: S14 File — (PDF) [file pone.0255326.s014.pdf]
