# Supplementary material for: Collection, genotyping and virus elimination of cassava landraces from Tanzania and documentation of farmer knowledge
Source: PLoS One. 2021 Aug 17;16(8):e0255326. doi: 10.1371/journal.pone.0255326 (PMC8370617; doi:10.1371/journal.pone.0255326)

PONE-D-20-24321

Ferguson et al. Collection, Genotyping and Virus Elimination of Cassava Landraces from Tanzania and Documentation of Farmer Knowledge

Supplementary file S15. Minimum spanning network, with edges connecting multilocus genotypes (MLG) according to the minimum genetic distance between samples. This allows for reticulations i.e. nodes with identical genetic distances can be connected in a network, as opposed to a tree whereby pairwise nodes are only connected to one other node with the shortest distances, even if several nodes share the same minimum genetic distance.

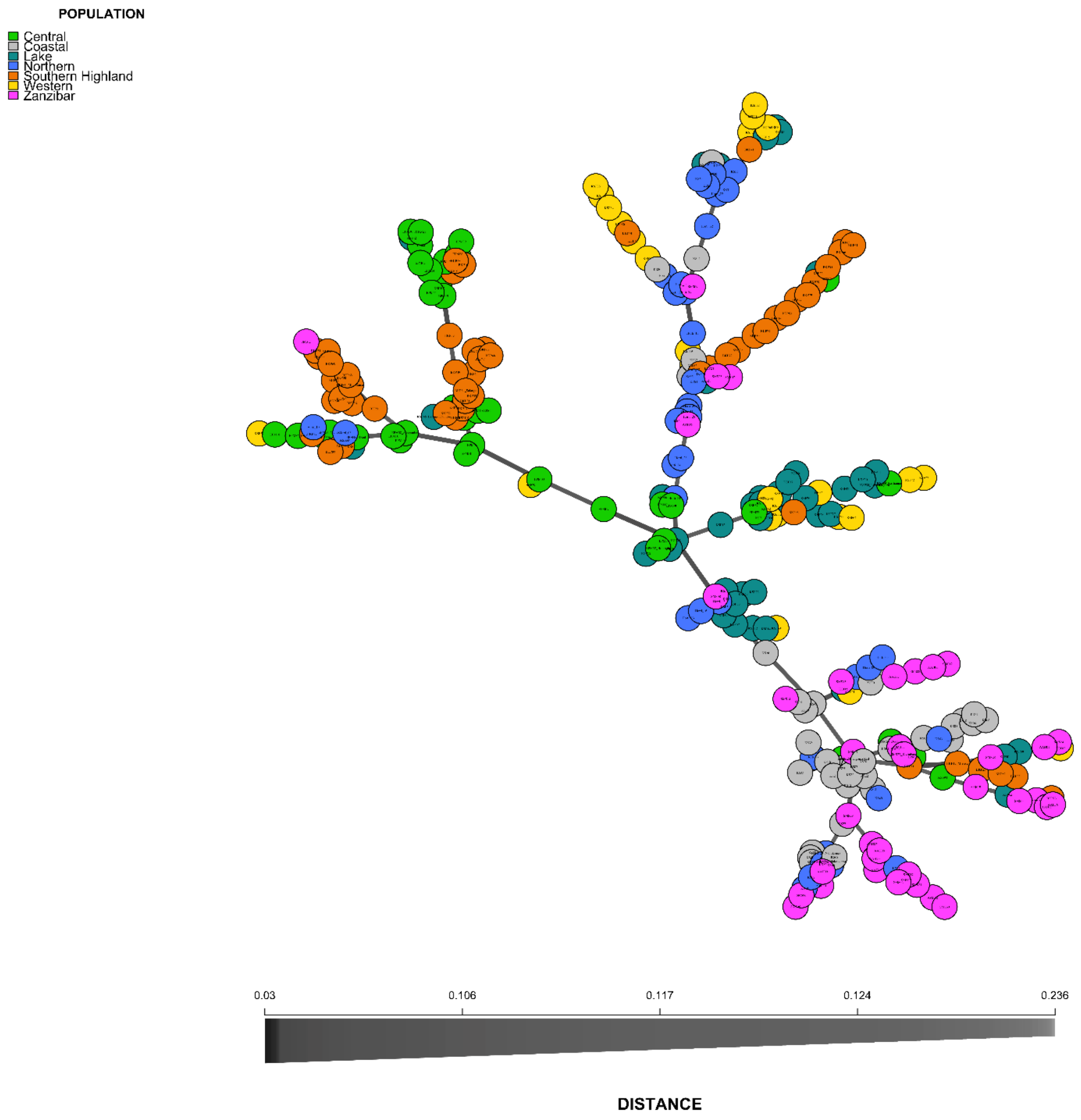

Supplement: S15 File — (PDF) [file pone.0255326.s015.pdf]
