# Supplementary material for: Collection, genotyping and virus elimination of cassava landraces from Tanzania and documentation of farmer knowledge
Source: PLoS One. 2021 Aug 17;16(8):e0255326. doi: 10.1371/journal.pone.0255326 (PMC8370617; doi:10.1371/journal.pone.0255326)
Supplement: S12 File — (PDF) [file pone.0255326.s012.pdf]

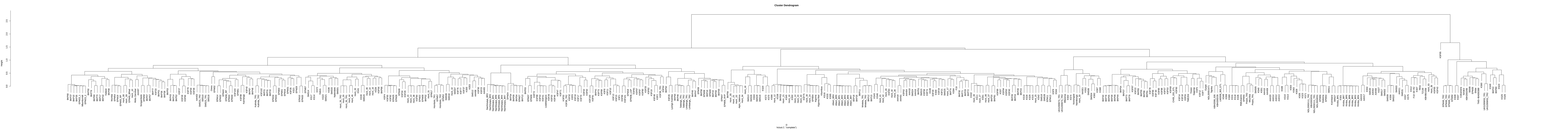
Cluster Dendrogram
Height
D
hclust (*, "complete")
